# Original quantitative research - Accidental substance-related acute toxicity deaths in older adults in 2016 and 2017: a national chart review study

**DOI:** 10.24095/hpcdp.44.3.03

**Published:** 2024-03

**Authors:** Jingru Helen Ha, Jacqueline Burt, Shane Randell, Amanda VanSteelandt

**Affiliations:** 1 Public Health Agency of Canada, Ottawa, Ontario, Canada; 2 Office of Drug Research and Surveillance, Health Canada, Ottawa, Ontario, Canada

**Keywords:** drug overdose, opioid overdose, mortality, older adults, substance use, Canada, acute toxicity

## Abstract

**Introduction:**

Limited research exists on substance-related acute toxicity deaths (ATDs) in older adults (≥60 years) in Canada. This study aims to examine and describe the sociodemographic characteristics, health histories and circumstances of death for accidental ATDs among older adults.

**Methods:**

Following a retrospective descriptive analysis of all coroner and medical examiner files on accidental substance-related ATDs in older adults in Canada from 2016 to 2017, proportions and mortality rates for coroner and medical examiner data were compared with general population data on older adults from the 2016 Census. Chi-square tests were conducted for categorical variables where possible.

**Results:**

From 2016 to 2017, there were 705 documented accidental ATDs in older adults. Multiple substances contributed to 61% of these deaths. Fentanyl, cocaine and ethanol (alcohol) were the most common substances contributing to death. Heart disease (33%), chronic pain (27%) and depression (26%) were commonly documented. Approximately 84% of older adults had contact with health care services in the year preceding their death. Only 14% were confirmed as having their deaths witnessed.

**Conclusion:**

Findings provide insight into the demographic, contextual and medical history factors that may influence substance-related ATDs in older adults and suggest key areas for prevention.

**Highlights**

- Males 60 years or older had a higher mortality rate due to accidental acute toxicity than females.
- When a pharmaceutical substance contributed to accidental acute toxicity death, 61% of older adults had a prescription for the substance. At least one nonpharmaceutical substance contributed to 43% of accidental older adult acute toxicity deaths.
- Multiple substances contributed to most of the accidental deaths (61%), with fentanyl, cocaine and ethanol (alcohol) most often contributing to death.
- Almost three-quarters of the older adults who died of acute toxicity had accessed health care in the year preceding death.

## Introduction

Substance-related acute toxicity deaths (ATDs) are an ongoing public health issue in Canada. At the national level, data on substance-related ATDs in specific subpopulations, for example, individuals aged 60 years and older, are limited. What is known is that people in this age group made up 9% to 11% of apparent opioid toxicity deaths from 2016 to 2022 and 8% to 10% of apparent stimulant toxicity deaths from 2018 to 2022.^1^ This study aims to better understand the characteristics of people aged 60 years and older who died of acute toxicity as well as the substances involved and circumstances of their deaths.

Several biomedical factors put older adults at high risk for acute toxicity: pharmacokinetic changes with age include increased body fat that prolongs the half-life of fat-soluble drugs (e.g. diazepam); decreased body water, which increases concentrations of water-soluble drugs (e.g. ethanol); reduced metabolism, which can lead to higher steady-state concentrations of some drugs (e.g. alprazolam); and reduced excretion of drugs (e.g. morphine) due to decreased cardiac output and kidney function.^2^ Very few clinical studies have been conducted to examine changes in pharmacokinetics and pharmacodynamics as people age, although adverse drug reactions are known to include increased mortality.^2^

For opioids in particular, the expected efficacy and side effects may change as regular biological functions change with age.^3^ Changes in drug distribution and impairment of metabolism and elimination of drugs can increase the potency, duration of action, and side effects of opioids.^3^ Disparities in drug distribution between older and younger adults may be due to decreases in digestion time and increases in gastric pH caused by medications commonly used by older people, as well as increases in adipose tissue combined with decreases in overall body mass and water volume.^3^

Older adults are more likely to have multiple prescriptions for multiple conditions, including for opioids for pain management,^4^,^5^ which increases the likelihood of incorrect ingestion of dosages as well as of errors in prescribing.^6^-^8^ A population-based cohort study from Ontario, Canada, found that adults older than 45 years and women were more likely to have an active opioid prescription at the time of death than adults younger than 25 years and men.^9^

Some older adults also use substances for relief of unmet medical or psychological needs or non-medical reasons. For example, among people aged 65 and older who consumed cannabis for medical and non-medical reasons, 72% did so for pain-related reasons, 29% to help with problems sleeping and 12% to manage anxiety or depression.^10^ About 6.1% of people aged 60 to 75 years reported using illicit substances in the past year and 7.6% reported high-risk alcohol use (one or more occasions of drinking 5 or more drinks or of drinking more than 10 (females) or 15 (males) drinks in the last week).^11^ Using other substances while taking prescription medications could increase the risk of acute toxicity events. Substance use can also lead to or escalate medical conditions common in older age, such as diabetes, cardiovascular disease or lung conditions,^12^-^14^ and these medical conditions could make older adults more susceptible to acute toxicity and at higher risk of death after an acute toxicity event.^15^-^17^

Because social isolation and stigma can also affect substance use among older adults, opportunities to modify risk of use may arise through social and structural interventions.^18^,^19^ For example, as a result of increasing medical concerns and prescriptions with age, older adults have a higher usage of health care services.^20^ Points of contact with health care services could be used as opportunities for substance use and mental health interventions, particularly for older adults with less social support.

Taken together, the medical histories and sociodemographic characteristics of older adults may increase the risk of accidental acute toxicity. This study examined accidental ATDs and sought, at the national level, to (1) describe the characteristics of older adults who died from acute toxicity and compare them with those of older adults in the general Canadian population; (2) characterize the substances contributing to ATDs in older adults as well as their origins (pharmaceutical vs. nonpharmaceutical) and sources (e.g. prescribed vs. diverted prescriptions); (3) explore the prevalence of physical and mental health conditions; and (4) describe social and environmental circumstances of ATDs among older adults.

## Methods

***Ethics statement***

This study was reviewed and approved by the Public Health Agency of Canada Research Ethics Board (REB 2018-027P), the University of Manitoba Health Research Ethics Board (HS22710) and Newfoundland and Labrador Health Research Ethics Board (20200153).

***Study design***

We used a retrospective, population-based, cross-sectional chart review design to explore the relationship between accidental ATDs and older adult sociodemographic risk factors, prescription and medical history, circumstances of death and toxicological findings. All coroner and medical examiner files involving substance-related ATDs that occurred in Canada between 1 January 2016 and 31 December 2017 were reviewed and abstracted into a common database. Data abstractors were trained to abstract information according to a comprehensive study protocol that provided guidelines on case definition, data classification and how to describe certain data. Electronic databases were mapped to the study’s database variables. The study’s co-investigator team, who developed the study protocol and reviewed all analysis projects using the dataset, includes people with expertise in death investigations, toxicology, public health, harm reduction and Indigenous health research and with lived experience of substance use. A detailed explanation of the study design and abstraction process21 and a general summary of the dataset22 have been reported elsewhere.

***Study population***

Our study included all people in Canada who died at age 60 years and older as a result of an accidental substance-related ATD. A substance-related ATD refers to any death that is a direct result of the administration of one or more exogenous substances that is a drug or alcohol.

A cut-off of age 60 years and older was used in accordance with literature on older adulthood;^23^-^25^ this was also supported by the natural breaks in the data we observed during exploratory data analysis. Depending on minimum cell sizes, stratification varied by 10-year age categories or no stratification (all older adults). For the 10-year age categories, individuals 80 years and older were combined into one age group due to smaller counts with increasing age.

***Analysis***

A descriptive summary of older adult characteristics was generated from chart review data. Dichotomous and categorical variables were reported as percentages. As death investigation files are not a complete record of a person’s life and information available in the files varies, it is important to note that these percentages are the minimum proportions of older adults that had a given characteristic. Further analyses investigated proportions and mortality rates for older adults who died of accidental acute toxicity and older adults in the general Canadian population. Data sources for the general Canadian population included the 2016–2017 Labour Force Survey^26^ and the 2016 Census profile.^27^ Chi-square tests for independence were conducted between age cohorts where minimum cell sizes permitted. In addition to examining older adults as a specific age group, we applied a sex-based approach to the analysis, minimum cell size permitting.^28^ All statistical analyses were carried out using statistical computing software R version 4.2.2 (R Foundation for Statistical Computing, Vienna, AT).^29^

Our analysis had four aims. The first aim was to compare older adults who died of accidental acute toxicity with older adults in the general Canadian population by demographic characteristics including age, sex, income and living arrangements. The second aim was to describe the most common substances and combinations of substances that contributed to death for older adults and explore the origin (pharmaceutical vs. nonpharmaceutical) and source (e.g. prescribed vs. diverted prescriptions) of these substances with an UpSet plot constructed using the ComplexUpSet package.^30^

The third aim was to examine and report on documented health conditions or symptoms, health service utilization, prescribed medications and history of substance use. Documented history of mental and physical health conditions or symptoms is based on any information available in the death investigation file, including medical records or witness statements (family or friends). Therefore, these conditions or symptoms are not necessarily clinical diagnoses, were not necessarily experienced at the time of death, and may not capture a person’s entire medical history. Prescription types refers to a medication class that was prescribed up to 6 months before death. Prescriptions associated with chronic pain were based on a framework for the most commonly prescribed medications for treatment of chronic pain and includes unspecified opioid prescriptions.^31^

The fourth aim of the analysis was to explore and describe the circumstances of ATDs: the location of death, the possibility of medical error, how often deaths were witnessed, how often naloxone was administered, and the most common modes of substance use.

To protect privacy, any cell that contained a count of less than 10 was suppressed and all counts were randomly rounded to base 3 (i.e. values had different chances of being rounded to nearest multiples of 3).^21^ Since table totals were independently rounded to base 3, the sum of values do not always equal the total. Any calculated percentages and crude rates were based on the rounded counts. Test statistics and exact *p* values are not provided to protect random rounding.

## Results

***Accidental acute toxicity deaths***

We identified 705 older adults who died of accidental substance-related acute toxicity between 1 January 2016 and 31 December 2017. The accidental ATD mortality rate was 4.3 per 100000 population for all older adults (Table 1). The accidental ATD mortality rate was highest for adults aged 60 to 69 years (7.1 deaths per 100000 population) and lowest for those aged 80+ years (0.6 per 100000 population). The mortality rate for males was approximately double that for females (6.0 vs. 2.8deaths per 100000 population).

**Table 1:** Distribution of characteristics among adults who died of accidental acute toxicity and among the general population, ≥60 years, and accidental mortality rates, Canada, 2016–2017

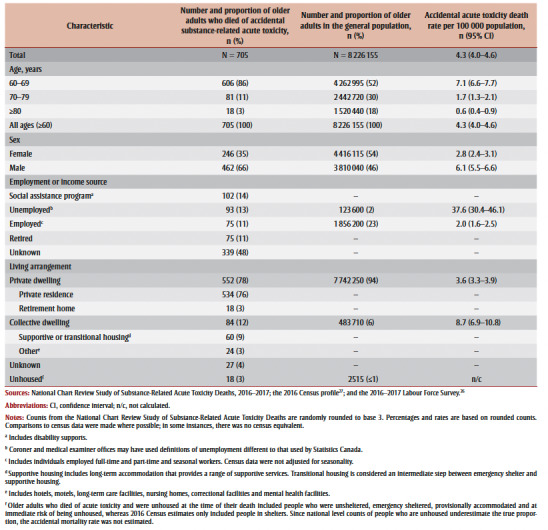

At 35%, the proportion of female older adults who died from acute toxicity was smaller than the proportion of female older adults in the overall population (54%) ( Table 1). Conversely, a higher proportion of male older adults died from acute toxicity (66%) than the proportion of male older adults in the overall population (46%). A higher proportion of adults aged 60 to 69 years died of acute toxicity (86%) than the proportion in the overall population (52%). The proportion of adults aged 70 to 79 years (11%) and 80 years and older (3%) was lower among those who died of acute toxicity than in the overall population (30% and 18%, respectively).

Of the older adults who died of accidental substance-related acute toxicity, a higher proportion was unemployed than the proportion of unemployed older adults in the overall population (13% vs. 2%) (Table 1). The accidental mortality rate for older adults was much higher for those who were unemployed than for those who were employed (37.6 vs. 2.0 deaths per 100000 population). No comparison statistic for those who were retired was available. Older adults living in a private dwelling had a lower accidental mortality rate than those in collective dwellings (3.6 vs. 8.7 deaths per 100000 population).

***Substances contributing to accidental death***

The most common substances that contributed to death in older adults were fentanyl (27%; 189/705), cocaine (27%; 189/ 705), ethanol (alcohol; 23%; 165/705) and morphine (15%; 105/705) (Table 2). At least one nonpharmaceutical substance (unregulated drugs and substances not intended for human use, such as industrial or household chemicals or veterinary medications, and not including ethanol [alcohol]) contributed to the death of 43% (300/705) of older adults who died of accidental acute toxicity, and at least one pharmaceutical substance contributed to the death of 49% (342/705). Substances of both nonpharmaceutical and pharmaceutical origin contributed to 10% of deaths (data not shown). When a pharmaceutical substance contributed to a person’s death, at least 60% (204/342) of the time that substance had been prescribed to that person.

**Table 2:** Specific substances most commonly contributing to accidental acute toxicity deaths of adults, ≥60 years, their origin and the contributions of other substances, Canada, 2016–2017 (N = 705)

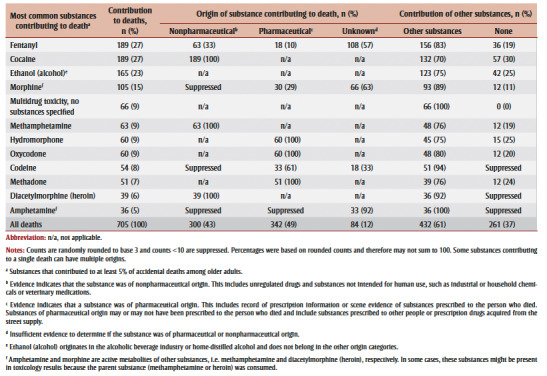

**Figure 1:**
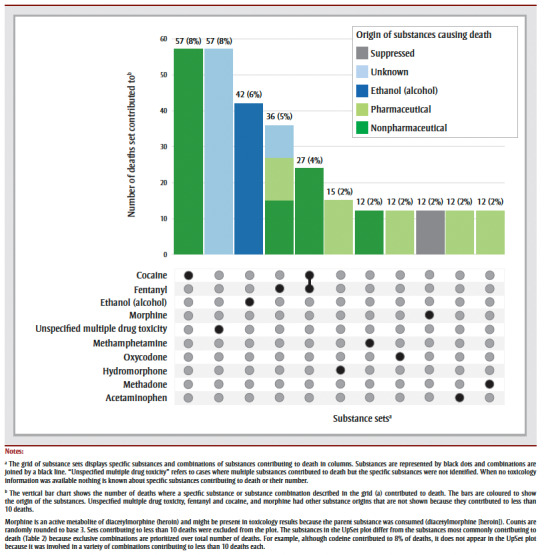
UpSet plot of the top substances and substance combinations contributing to accidental acute toxicity deaths of adults, ≥60 years, by origin, Canada, 2016–2017 (N = 705)

Multiple drug toxicity due to unspecified substances (8% of deaths; 57/705) and fentanyl and cocaine (4%; 27/705) were the most common substance combinations contributing to death (Figure 1). Multiple substances contributed to 61% (432/705) of older adult deaths, but only the two substance combinations mentioned occurred in more than 10 deaths. The top three substances that contributed to deaths on their own (without a contribution from other substances) were cocaine (8% of deaths; 57/705), ethanol (6%; 42/705) and fentanyl (5%; 36/705).

Most of the substance sets had a single origin, except for fentanyl, fentanyl and cocaine combined, and morphine ( Figure 1). Fentanyl that contributed to deaths alone was pharmaceutical (30% of deaths) and nonpharmaceutical (42% of deaths) in origin. When fentanyl and cocaine were combined, they were mainly both nonpharmaceutical in origin, with fewer than 10 deaths due to combined pharmaceutical and nonpharmaceutical origin (not shown due to small counts). Morphine had a mix of pharmaceutical and unknown origins (origins not shown due to small counts).

***Health history***

Several health conditions or symptoms were documented for 10% or more of the older adults who died of accidental acute toxicity (Table 3). Around one-quarter (26%; 186/705) had current or past depression. Prevalence of current or past anxiety disorder, alcohol use disorder and substance use disorder other than alcohol was about the same, at 14% (96–102/705). At least 17% (123/705) had two or more of these mental health conditions or symptoms.

**Table 3:** Documented health histories of adults, ≥60 years, who died of accidental acute toxicity, Canada, 2016–2017 (N = 705)

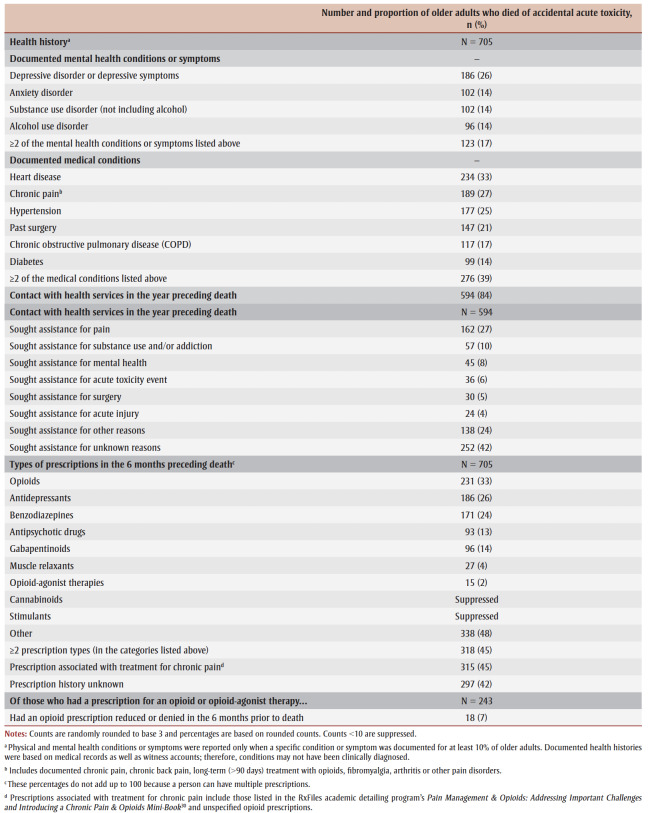

About one-third (33%; 234/705) of older adults who died had a history of heart disease, 27% (189/705) had a history of chronic pain and 21% (147/705) had a past surgery. Comorbidity was common; 39% (276/705) had two or more of these documented health conditions. The majority (84%; 594/705) had contact with health services in the year preceding their death. Of those who had contact with health services, 27% (162/594) had sought assistance for pain-related reasons.

Many older adults who died of accidental acute toxicity had prescriptions for opioids (33%; 231/705), antidepressants (26%; 186/705) and benzodiazepines (24%; 171/705). Almost half (45%; 318/705) had multiple prescription types and 45% (315/705) had at least one prescription associated with chronic pain treatment.

***Circumstances of death***

Of all accidental older adult ATDs, fewer than 10 had evidence of a medication error, that is, that a prescription medication was mistakenly consumed (e.g. not realizing what it was, or forgetting that a dose had been taken already and consuming more), incorrectly dispensed drugs at a pharmacy or incorrectly administered drugs in a hospital.

Only 14% (96/705) of lethal acute toxicity events were definitely witnessed, and 17% (120/705) of deaths were potentially witnessed by someone who might have been able to respond (it was unclear if the person was still alive or deceased when found) (Table 4). In 39% (273/705) of cases, the person had already died when they were found, and there was no evidence that the acute toxicity event had been witnessed. Adults aged 80 years and older had their acute toxicity event witnessed significantly more often than those aged 60 to 69 years (chi-square test, *p*<0.05; data not shown).

**Table 4:** Circumstances of death of adults, ≥60 years, who died of accidental acute toxicity, Canada, 2016–2017 (N = 705)

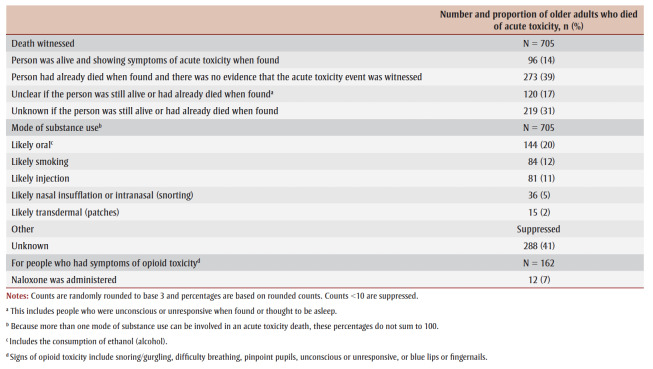

The mode of substance use was unknown for 41% (288/705) of older adults. The most commonly reported mode of substance use was oral (20%; 144/705), smoking (12%; 84/705) and injection (11%; 81/705). Of older adults who died and were observed to have signs of opioid toxicity (i.e. snoring/gurgling, difficulty breathing, pinpoint pupils, unconscious or unresponsive, or blue lips or fingernails), only 7% (12/162) were administered naloxone.

## Discussion

This study examined accidental ATDs among adults aged 60 years and older that occurred between 1January 2016 and 31 December 2017 in Canada. ATDs occurred more often in those aged 60 to 69 years, males and those who were unemployed. These findings align with previous research that suggests that the sex distribution of opioid deaths in Canada is disproportionately represented in males.^32^,^33^ Unemployment has also previously been identified as a risk factor for higher-risk use of opioids and death.34,35 Coroner and medical examiner offices may use different definitions for unemployment than Statistics Canada; therefore, this finding should be interpreted with caution.

Fentanyl and cocaine contributed to most of the accidental ATDs among older adults. The origin of the fentanyl was a mixture of nonpharmaceutical (illegal) and pharmaceutical fentanyl. Fentanyl was the sixth and fourth most prevalent illegal substance seized by law enforcement organizations across Canada and tested by Health Canada’s Drug Analysis Service (DAS) in 2016 and 2017, respectively.^36^,^37^ However, at least one-third of the fentanyl contributing to the ATDs of older adults was of pharmaceutical origin. Most of the time, fentanyl was one of multiple substances contributing to death. Nonpharmaceutical fentanyl is commonly used to contaminate other substances and is deliberately combined with other substances.^38^ The high proportion of ATDs associated with fentanyl in combination with other substances could also be related to the higher likelihood of older adults having multiple prescriptions.^6^,^39^

Of the substances most commonly contributing to death in older adults—fentanyl, cocaine, ethanol and morphine— only cocaine has entirely nonpharmaceutical origins. In 2016 and 2017, cocaine was the most prevalent controlled substance, after cannabis, to be seized by law enforcement in Canada.^36^,^37^ Cocaine use by older adults is often under-screened and unrecognized, although it is a growing concern.^40^,^41^ Cocaine use can contribute to the development of heart disease and cause death through its acute effects on the cardiovascular system^42^; it is noteworthy that 33% of older adults who died of acute toxicity had a documented history of heart disease.

Multiple substances contributed to the majority of accidental older adult deaths; however, only one combination, fentanyl and cocaine, occurred in more than 10 deaths. This suggests that a wide variety of substance combinations contribute to the death of older adults. Substances of nonpharmaceutical and pharmaceutical origin both commonly contributed to accidental deaths among older adults, and substances of both origins contributed to at least 11% of the deaths. Programs and policies that reduce the harms of the illegal drug supply and pharmaceuticals would both be beneficial for this population.

Where substances of pharmaceutical origin contributed to the accidental ATD, the majority had been prescribed to the person who had died (61%). This finding suggests a need to improve assessment identifying inappropriate prescribing or potentially harmful use of or dependence on prescribed substances. In this study, we found that 45% of older adults who died were known to have had two or more known prescription types in their medical history. True rates are likely higher given potential under-ascertainment of data on prescribed substances throughout the data abstraction process. For comparison, some degree of polypharmacy has previously been found in 65% of older adults.^4^ In this study and the general population, many older adults have multiple prescriptions to manage their medical conditions; improving our understanding of the risks of polypharmacy and managing and communicating these risks is also important. A 2016 systematic review of opioid and benzodiazepine misuse in older adults suggests that this is a growing concern in an aging population.^43^ The authors suggest there is an increasing need for education of primary care providers on risk-reducing prescription practices and for policy maker and stakeholder engagement in prescription monitoring programs.^43^ Where pharmaceutical substances and polypharmacy are concerned, ATDs due to medication errors were rare, although this may have been underestimated because of a lack of documentation in coroner and medical examiner files or the absence of witnesses able to notice errors in dosage.

Depressive disorders or symptoms of depressive disorders were documented for one in four older adults who had died due to accidental acute toxicity, and anxiety disorder was documented for more than one in ten. Older adults with severe depression symptoms are more likely to be taking high-potency opioid medications.^44^ Social isolation, depression and anxiety increased during the COVID-19 pandemic,^45^ and these have been linked with higher-risk substance use among older adults.^18^,^46^ These factors, combined with decreased access to harm reduction and other health services during the pandemic, may have put older adults at greater risk of ATDs since our study period.

To reduce the risk of opioid overdose, people using substances should not use alone and should have a naloxone kit available.^46^ A substantial portion (at least 38%) of accidental overdoses occurred when the older adults were alone, and only 7% of the older adults who showed signs of opioid toxicity received naloxone.^47^ Literature on the relationship between social isolation indicators, including living alone, and substance use is divided, with some studies suggesting that social isolation is not associated with overdose death and others suggesting it is.^48^-^51^ According to the British Columbia Centre for Disease Control *Take Home Naloxone Program Report*, 3.7% of clients who received naloxone kits in 2017 were aged 60 years and older,^52^ while people in that age group made up 7% of illicit drug toxicity deaths in British Columbia in 2016 and 2017.^53^ Improved access to harm reduction services and social supports for older adults who use substances could prevent ATDs.

Contact with health services presents an opportunity to screen older adults for social supports, inquire about high-risk substance use behaviours and provide naloxone kits.^51^,^54^ As 72% of the older adults who died had accessed health care in the year preceding their death, health care visits may present opportunities to identify potential issues in prescribing and substance use and address them. About one in five of these consultations with health care services were pain-related and could involve assessments of and discussions about pain management, including prescribed medications and relief sought from other substances, as well as alternative approaches to alleviate pain.

While deaths due to accidental acute toxicity are less common among older adults than among younger adults and most deaths in this age group are due to chronic diseases such as heart disease, cancer and stroke,^55^ given the preventable nature of ATDs, the risk factors identified in this study have practical implications for interventions that may reduce the burden of acute toxicity mortality in older adults in Canada.

***Strengths and limitations***

Data for this study were collected from coroner and medical examiner files across all the provinces and territories by trained data abstractors. Prior to and during the collection period, data abstractors completed intra-rater and inter-rater reliability testing to ensure reliable data collection. However, the information available differed across individual files and coroner and medical examiner offices in terms of investigation processes, forms and toxicological testing. The files are administrative in nature and exist to document investigations into cause and manner of death; they are not a complete record of a person’s life. This may have resulted in underreporting of certain factors where data were unavailable, and therefore the proportions in this study represent the minimum number of older adults who died of acute toxicity and had a given characteristic.

Additional factors aside from those reported in this study may have contributed to the risk of accidental ATDs among older adults. For example, First Nations people have been disproportionately impacted by the overdose crisis.^32^ Although data about people with diverse gender identities are sparse, a number of interrelated factors are known to put them at high risk of substance use-related harms.^56^ Also, this study examines deaths at the national level, but there may be differences in local contexts, including access to services and to illegal drug markets, that affect risk of death. Some of these factors and their intersectionality will be explored in future work.

In addition, limited comparisons could be drawn between ATD data in this study and other data sources because of the differences in definitions of variables. In particular, it is unclear if coroner and medical examiner offices defined unemployment in the same way as was done by Statistics Canada in the Labour Force Survey. Despite these limitations, this study provides an in-depth descriptive nationwide review of Canadian coroner and medical examiner files for ATDs in older adults.

## Conclusion

This is the first nationwide Canadian study to use coroner and medical examiner files to examine the characteristics of older adults who died from acute toxicity as well as the substances involved and circumstances of their deaths. As such, it provides a baseline for comparison as the overdose crisis changes with time. The drivers of acute toxicity in older adults are multifaceted and unique to this population. Heart disease, depression, chronic pain and polypharmacy were highly prevalent among older adults who died of accidental acute toxicity; however, high levels of contact with health services provides opportunities for intervention to better understand motivations for substance use and reduce the risk of potential harms.

## Acknowledgements

We would like to thank our collaborators at the offices of chief coroners and chief medical examiners across Canada for providing access to their death investigation files. We would also like to acknowledge Brandi Abele, Matthew Bowes, Songul Bozat-Emre, Jessica Halverson, Dirk Huyer, Beth Jackson, Graham Jones, Fiona Kouyoumdjian, Jennifer Leason, Regan Murray, Erin Rees, Jenny Rotondo and Emily Schleihauf for their contributions in developing the national chart review study on substance-related ATDs. We are grateful for the contributions of people with lived and living experience of substance use who have supported the chart review study at multiple stages.

## Funding

This study was funded by the Public Health Agency of Canada. 

## Conflicts of interest

The authors report no conflicts of interest.

## Authors’ contributions and statement

JB – Conceptualization, writing – original draft, writing – review & editing.

SR – Conceptualization, writing – original draft, writing – review & editing.

AV – Conceptualization, data curation, supervision, writing – original draft, writing – review & editing.

JH – Conceptualization, data curation, project administration, writing – original draft, writing– review & editing.

Opinions and conclusions in this report are the authors’ own and not endorsed or approved by data providers or funders. The content and views expressed in this article are those of the authors and do not necessarily reflect those of the Government of Canada.
